# Energy balance model of mass balance and its sensitivity to meteorological variability on Urumqi River Glacier No.1 in the Chinese Tien Shan

**DOI:** 10.1038/s41598-019-50398-4

**Published:** 2019-09-27

**Authors:** Yanjun Che, Mingjun Zhang, Zhongqin Li, Yanqiang Wei, Zhuotong Nan, Huilin Li, Shengjie Wang, Bo Su

**Affiliations:** 1 Department of Geography Science, Yichun University, Yichun, 336000 Jiangxi China; 2 College of Geography and Environmental Science, Northwest Normal University, Lanzhou, 730070 Gansu China; 3 State Key Laboratory of Cryospheric Sciences, Northwest Institute of Eco-Environment and Resources/Tianshan Glaciological Station, Chinese Academy of Sciences, Lanzhou, 730000 Gansu China; 4 Key Laboratory of Remote Sensing of Gansu Province, Northwest Institute of Eco-Environment and Resources, Chinese Academy of Sciences, Lanzhou, 730000 Gansu China; 5 Jiangsu Center for Collaborative Innovation in Geographical Information Resource Development and Application, Nanjing Normal University, Nanjing, 210023 Jiangsu China; 6 State Key Laboratory of Earth Surface Processes and Resource Ecology, Beijing Normal University, Beijing, 100875 China

**Keywords:** Cryospheric science, Hydrology

## Abstract

Energy exchanges between atmosphere and glacier surface control the net energy available for snow and ice melt. Based on the meteorological records in Urumqi River Glacier No.1 (URGN1) in the Chinese Tien Shan during the period of 2012–2015, an energy-mass balance model was run to assess the sensitivity of glacier mass balance to air temperature (*T*), precipitation (*P*), incoming shortwave radiation (*S*_in_), relative humidity (*RH*), and wind speed (*u*) in the URGN1, respectively. The results showed that the glacier melting was mainly controlled by the net shortwave radiation. The glacier mass balance was very sensitivity to albedo for snow and the time scale determining how long the snow albedo approaches the albedo for firn after a snowfall. The net annual mass balance of URGN1 was decreased by 0.44 m w.e. when increased by 1 K in air temperature, while it was increased 0.30 m w.e. when decreased by 1 K. The net total mass balance increased by 0.55 m w.e. when increased precipitation by 10%, while it was decreased by 0.61 m w.e. when decreased precipitation by 10%. We also found that the change in glacier mass balance was non-linear when increased or decreased input condition of climate change. The sensitivity of mass balance to increase in *S*_in_, *u*, and *RH* were at −0.015 m w.e.%^−1^, −0.020 m w.e.%^−1^, and −0.018 m w.e.%^−1^, respectively, while they were at 0.012 m w.e.%^−1^, 0.027 m w.e.%^−1^, and 0.017 m w.e.%^−1^ when decreasing in those conditions, respectively. In addition, the simulations of coupled perturbation for temperature and precipitation indicated that the precipitation needed to increase by 23% could justly compensate to the additional mass loss due to increase by 1 K in air temperature. We also found that the sensitivities of glacier mass balance in response to climate change were different in different mountain ranges, which were mainly resulted from the discrepancies in the ratio of snowfall to precipitation during the ablation season, the amount of melt energy during the ablation season, and precipitation seasonality in the different local regions.

## Introduction

Glacier play a crucial role in streamflow regimes in the arid regions of Central Asian, especially during the summer with little precipitation, as meltwater from the glacier is released when other sources (e.g. precipitation and snowmelt) are depleted^1–3^. For example, the glacier meltwater is the important freshwater to supply for resident live and agricultural irrigation in Tien Shan and its around regions, i.e., Kyrgyzstan, Kazakhstan, Uzbekistan, Turkmenistan, and Xinjiang of China^4,5^. As global warming, the glaciers in Tien Shan were widely retreated since the mid-nineteenth century, and the retreated trend has been accelerated since the 1970s^6–10^. In addition, the glaciers showed the different behaviors under the global warming, that is, corresponding to the different respond regimes of glaciers to climate change. For example, the glacier retreat in Eastern Tien Shan was more significant than that in Western Tien Shan^10–12^.

The environment of glacierized regions and glacier-melt patterns were changed with global warming, such as the changes in air temperature, precipitation, and their contribution to glacial runoff. Some signals in glacier melting were used to understand the climate change, in other words, a glacier’s climate sensitivity can be described in terms of the energy or mass balance response to a change in meteorological conditions^13,14^. The air temperature and precipitation, which were widely used to construct the function of glacier mass balance in response to climate change. Although the simply models (e.g., Temperature-Index models) had a good skill at estimating seasonal melt and were used to assess the sensitivities of glacier mass balance to the air temperature and precipitation^15–21^, they were missing much of the physics that govern melt^17,22,23^. Temperature-index or enhanced temperature-index models, which showed the over sensitive to the temperature. Because their melt process of glaciers were directly dependent on the air temperature, without considering the impacts of shift in other variables, such as radiation flux, rainfall flux, wind, humidity, or cloud cover^23,24^.

The Energy Mass Balance model is equipped with more-sophisticated physical processes^15,22,24,25^. The models can compute the component of mass balance from each relevant energy fluxes at the glacier surface, which is important to understand the interaction between glacier melt and climate change. For the continental glaciers, one study found that net radiation was the main source of melt energy, accounting for around 70–80% of the total melt energy^18,26–28^. The shortwave radiation provided the principal energy source, which was not directly dependent on the air temperature. The sensible and latent heat fluxes, however, were important energy to glacier melt, especially for those glaciers in maritime and tropical environments^29,30^. In addition, a study of Kersten Glacier found that the majority of the mass loss (∼65%) was due to sublimation (direct conversion of snow/ice to water vapour), with melting of secondary importance^31^. To assess the performance of the models with different melt regimes, an experiment was conducted by running the five different melt models^32^, including the classical Temperature-Index model, Temperature-Index model of Hock^15^, an Enhanced Temperature-Index model, a simplified Energy-Balance Model, and an Energy-Balance Model. Their simulations showed that the biases between measurements and simulations of temperature-index model were significantly increasing as time increasing, while the running of energy mass balance models showed the relatively more stable.

**Figure 1:**
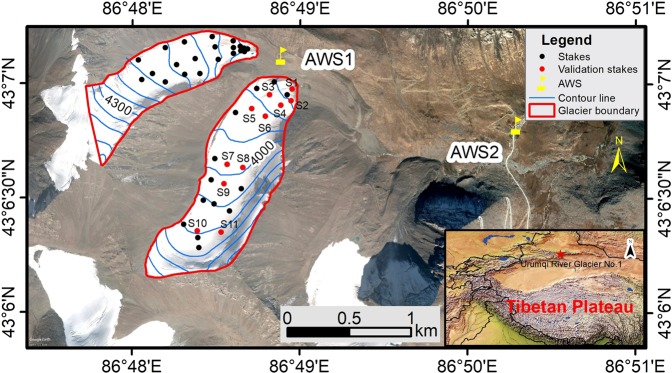
Observation sites in the Urumqi River Glacier No.1. In plot, glacier boundary (red line) indicates the boundary of Urumqi Glacier No.1 in 2012, AWS1 and AWS2 denote the automatic weather station closest to the glacier, and the stakes and validation stakes were the *in situ* stakes of mass balance. In addition, the interval elevation of contour line (blue line) was 50 m. The raster map data for High Asia in the subplot was downloaded from http://www.naturalearthdata.com/. The satellite image, which was provided by DigitalGlobe, was annotated with the positions of stakes, meteorological stations and the contour lines of glacier surface in Google Earth Pro version 7.3 (https://www.google.com/earth) and exported. The final map of glacierized region was created by ArcMap version 10.2 (http://desktop.arcgis.com/en/arcmap/) by adding necessary map elements to the exported and spatially-registered image.

Urumqi River Glacier No.1 (URGN1), which is located at the headwater of Urumqi River, eastern Tien Shan (also called as Chinese Tien Shan). To calculate mass balance of the glacier, first measurement was carried out by Tianshan Glaciological Station of the Chinese Academy of Sciences in 1958/59 (denoted by 1959), with glacier area of 1.91 km^2^ and glacier length of 2.2 km^33^. In 1993, the glacier was entirely separated into two branches due to glacier retreat, i.e., east branch and west branch glacier (Fig. 1). Air temperature and precipitation were used to assess the sensitivities of glacier mass balance, which were only simulated in 1998 using the simple Temperature-Index models^34,35^. According to the latest survey in 2012, the glacier area decreased to 1.59 km^2^, meanwhile, 45 sites (including 42 measurement stakes of mass balance and 3 snow pits) were measured to understand the annual mass balance of URGN1^33^. In addition, elevation of the glacier surface ranged from 4484 m a.s.l. to ~3800 m a.s.l. Local environment and glacier melt pattern were changed due to climate change and glacier retreat.

URGN1 has the longest records of glacier observation in China (https://wgms.ch/), which is only one Chinese glacier of ‘reference glacier’ that was published by World Glacier Monitoring Service (WGMS). It is also important to understand the glaciers melting, hydrological cycle, and the mechanism between glacier and climate change in Tien Shan. However, we still don’t know which component of energy balance dominated the URGN1 melt-pattern and this point needs to be clear as soon as possible. It was still no clear how the glacier sensitivity was in response to climate change. Herein, we forced on the distributed Energy-Mass Balance model in order to understand the sensitivity of the glacier mass-balance to climate change. In this paper, our aims mainly focused on the two points: (I) Glacier melting of URGN1 was mainly dominated by which meteorological elements; (II) How much increased precipitation can be compensated to the glacier mass loss resulted from the glacier melt due to the increase of 1 K in air temperature, which would greatly help us to understand the glacier change in the future. In addition, the mass balances of 11 stakes (red points in Fig. 1) were used to validate the running of Energy Mass Balance model. Those stakes had the relative good continuity in terms of *in situ* mass balance during the simulated period, which located at the east branch glacier.

## Results

### Climate change

Ice cores were used to understanding palaeoclimate series, because they kept some important natural information, such as, local temperature, precipitation, volcanic, moisture source conditions^36^. To understand the palaeoclimate in the study region, an experiment of studying the relationship between stable oxygen isotope with air temperature and precipitation respectively was implemented from June 1995 to June 1996. Based on the analyses of stable oxygen isotope in precipitation, snow pits, and ice core samples, the results^37^ showed that the snow deposition process was destroyed by melting water percolation processes, the vapour movement within the snow packs, and redistribution of snowpacks by wind-blowing. The record of stable oxygen isotope (e.g., *δ*^18^O) in ice cores, therefore, was not used to reconstruct the paleoclimate environment (e.g., air temperature, precipitation, and wind) in the study region^37^.

**Figure 2:**
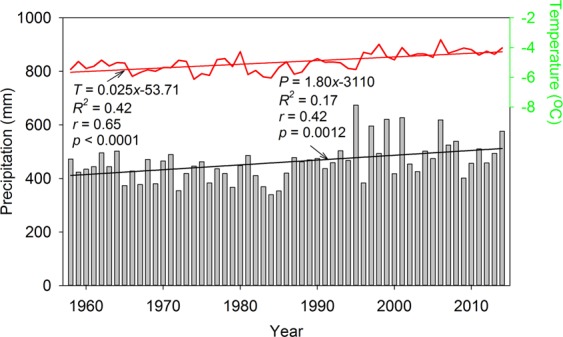
Changes of air temperature and precipitation recorded by Daxigou meteorological station (AWS2). In plot, gray bar denotes the precipitation, while the red line denotes the air temperature. In addition, *T* denotes the air temperature, *P* denotes the precipitation, *r* denotes the relative coefficient, *R*^2^ denotes the coefficient of determination to fit equation, and *p* denotes the significance level of statistic test.

Based on the records in automatic weather station 2 (AWS2), we found that the mean annual air temperature was −4.97 °C, and there was a significantly increased trend of 0.25 °C/decade (*p* < 0.0001) from 1958 to 2015 (Fig. 2). The linear regression equation of the changed trend in air temperature was *T* = 0.025*x* − 53.71 (*T* denotes the air temperature in unit °C/yr, *x* denotes the variable year), the coefficient of determination *R*^2^ was 0.42, the relative coefficient *r* was 0.65, and significance level of statistic test *p* < 0.0001. For annual precipitation, the mean annual value was at 461.64 mm and increased by 18 mm/decade. The linear regression equation of precipitation was *P* = 1.80*x* − 3110 (*P* denotes the annual precipitation in unit mm/yr), and its static parameters were *R*^2^ = 0.17, *r* = 0.42, and *p* < 0.01. To further understand the glacier meteorological condition, we established the meteorological observation field in the front of URGN1 in summer of 2012 (i.e., AWS1 in Fig. 1). In the field, some meteorological elements were automatically monitored and recorded at every five minutes during the period of 2012–2015, including shortwave radiation, longwave radiation, air temperature, precipitation, wind speed, relative humidity, and so on. The precipitation was recorded by T-200B, and the air temperature was at 1.5 m height air temperature.

### Input of glacier-model and characteristic of meteorological element

Main data-requirement in the energy-mass balance model was meteorological data, which was often one station representative of study region. Meanwhile, a digital terrain model was necessary in order to associate boundary and derive grids. The meteorological data input to the model in this paper, which derived from the records of automatic weather station 1 (AWS1) (Fig. 1), including air temperature, relative humidity, wind speed, shortwave radiation, longwave radiation, and precipitation. The Digital Elevation Model (DEM) of ASTER GDEM is provided by Geospatial Data Cloud site, Computer Network Information Center, Chinese Academy of Sciences (http://www.gscloud.cn), whereas slope and aspect of the glacier were calculated using the DEM dataset. The initial conditions of running model also need the dataset of snow depth and glacier boundary, which derived from the measurement in the field and spatially interpolated to the raster data.

**Figure 3:**
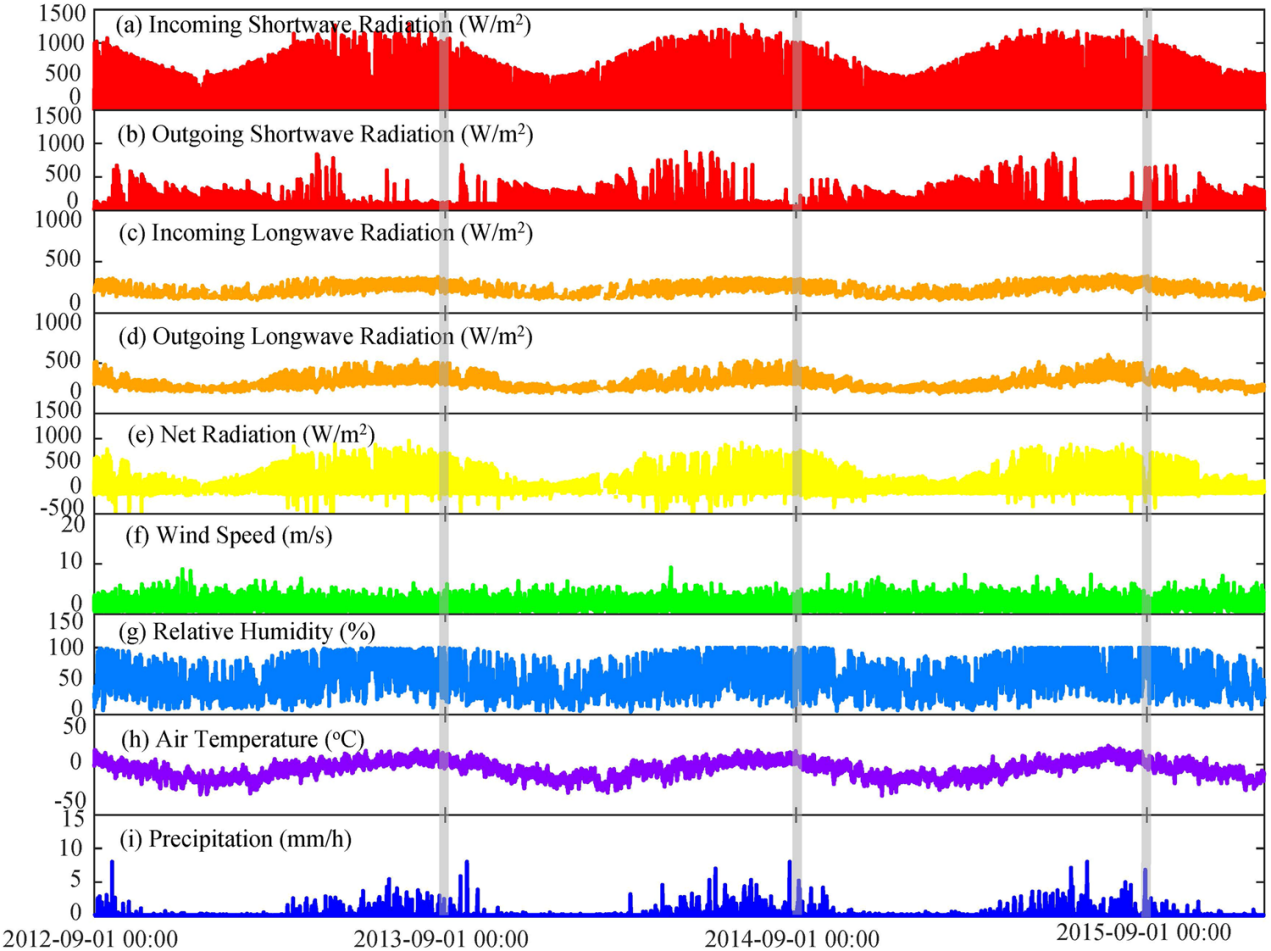
Meteorological variation recorded by Automatic Weather Station No.1 (AWS 1) in the field near to Urumqi River Glacier No.1.

The end August or early September, in general, is considered to the end of summer melting and the start of winter accumulation in Tien Shan. To conveniently initial the condition of snow depth on the glacier surface, 1st September in 2012 was regarded as the start time of model running. Thus, those meteorological data per hour were input to the model for simulating the mass balance of URGN1 during the period of 2012–2015 (Fig. 3), i.e., the incoming shortwave radiation (*S*↓), outgoing shortwave radiation (*S*↑), incoming longwave radiation (*L*↓), outgoing longwave radiation (*L*↑), wind speed (*u*), relative humidity (*RH*), air temperature (*T*_a_), and precipitation (*P*). It noted that the net radiation (*NR*) was calculated using the *S*↓, *S*↑, *L*↓ and *L*↑ (i.e., *NR* = *S*↓ − *S*↑ + *L*↓ − *L*↑). During the measured period, we also found that arithmetic mean of *S*↓ was at 173.57 W/m^2^ and ranged from 0 W/m^2^ to 1284 W/m^2^, arithmetic mean of *S*↑ was at 57.68 W/m^2^ and ranged from 0 W/m^2^ to 874 W/m^2^, arithmetic mean of *L*↓ was at 232.58 W/m^2^ and ranged from 125.97 W/m^2^ to 375.97 W/m^2^, and arithmetic mean of *L*↑ was at 303.34 W/m_2_ and ranged from 192.17 W/m^2^ to 583.40 W/m^2^. In addition, arithmetic means of *RH* and *u* were at 51.38% and 2.03 m/s, respectively.

### Sensitivity to parameter and characteristic of energy balance

To understand the air temperature pattern of the headwater region of Urumqi River, an observed experiment was implemented on the surface and at the terminus of URGN1 in summer of 2007, respectively. The results^38^ indicated that air temperature lapse rate was at −0.57 °C/100 m on the glacier surface, while it was at −0.59 °C/100 m from glacier front to ASW2. Here, the air temperature lapse rate (*T*_lapse_) of −0.58 °C/100 m (i.e., the arithmetic mean of the two temperature lapse rates) was used to calculate the air temperature of individual grid in our model. Temperature of glacial surface was assumed to 0 °C in this paper. If the precipitation increased by 22 mm/100 m with increasing altitude in this region, the altitude of maximum precipitation was deduced to be at 4050 m a.s.l^39^. Therefore, we divided the precipitation lapse rate as two different patterns in our simulations. After the model had been repeatedly run, calibrated, and linked the observation in the field, we found that the precipitation lapse rate (*P*_1_) was at +15% below the elevation of 4200 m a.s.l. in the study region, while it was at +8% above the elevation of 4200 m a.s.l (*P*_2_). Other initial conditions of model running were referenced from simulating of Reijmer and Hock^40^.

It is necessary to identify which parameters glacier model were more sensitive, herein, a local method was used in this paper that is called ‘one factor at a time’ (OAT) changes in parameters^41^. This method was widely used to understand the glacier sensitivity in terms of changes in input parameters^42–46^. Disadvantage of the methods was not considered parameter interaction. However, easy to implement and modest computational demands were the largest advantage for the models^43^. In general, change of 5% for physical parameters was used to simulate how much variation in model results, which could reflect the sensitivity of model parameters.

**Figure 4:**
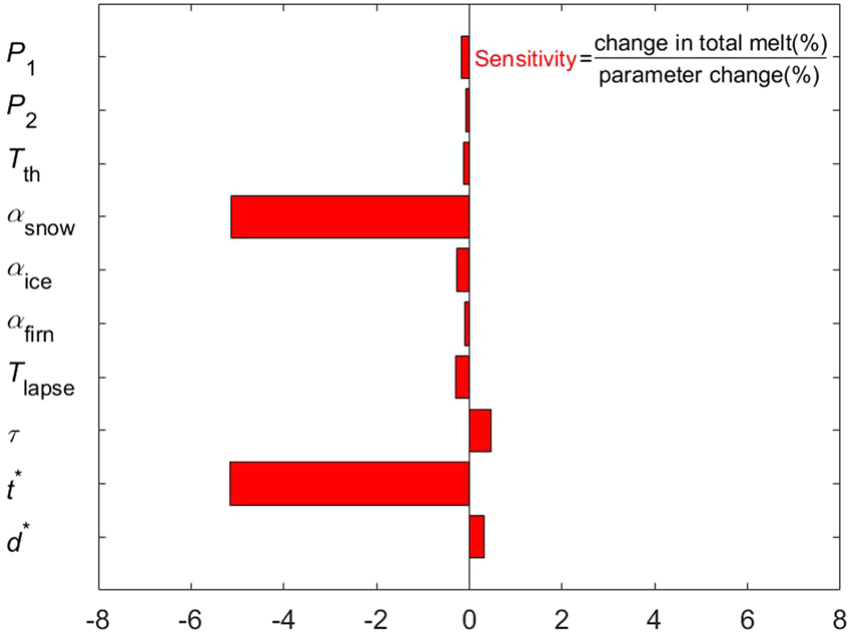
Sensitivities of mass-balance to glacial model parameters on the Urumqi Glacier No.1. In plots, *P*_1_ denotes precipitation lapse rate below 4200 m a.s.l., *P*_2_ denotes precipitation lapse rate above 4200 m a.s.l., *T*_th_ denotes air temperature threshold, *α*_snow_ denotes albedo for snow, *α*_ice_ denotes albedo for snow, *α*_firn_ denotes albedo for snow, *T*_lapse_ denotes the air temperature lapse rate, *τ* denotes the atmospheric transmissivity of clear-sky, t^*^ denotes the time scale determining how long the snow albedo decreases to the albedo for firn after a snowfall, and d^*^ denotes the characteristic scale for snow depth.

To find out which parameter largely impact on the output of simulating glacier melting, the parameter sensitivity test of OAT was conducted in this study (Fig. 4), and its sensitivity^42,43,46^ was defined as % melt change per % parameter change. The results showed that glacier melt had higher sensitive to parameters related to albedo for snow (*α*_snow_), the time scale (t^*^) determining how long the snow albedo close to the albedo for firn after a snowfall, atmospheric transmissivity of clear-sky (*τ*), characteristic scale for snow depth (d^*^), and air temperature lapse rate (*T*_lapse_). Albedo determines the available net shortwave radiation on the glacier surface, which directly influences the glacier surface evolution. Therefore, the sensitivity of the glacier to albedo-related parameters, especially *α*_snow_ and t^*^ were very higher than other parameters. The mass balance was also sensitive to *T*_lapse_ because the air temperature of grid cells calculated from the air temperature records of meteorological station. Sensitivity of the glacier mass change to other parameters were no more than 0.3, including precipitation lapse rates (*P*_1_ and *P*_2_), air temperature threshold (*T*_th_), albedo for ice (*α*_ice_) and firn (*α*_firn_), respectively. The simulations also presented that the glacier mass balance was more sensitivity to precipitation lapse rate below 4200 m a.s.l. (*P*_1_) than that above 4200 m a.s.l. (*P*_2_).

**Figure 5:**
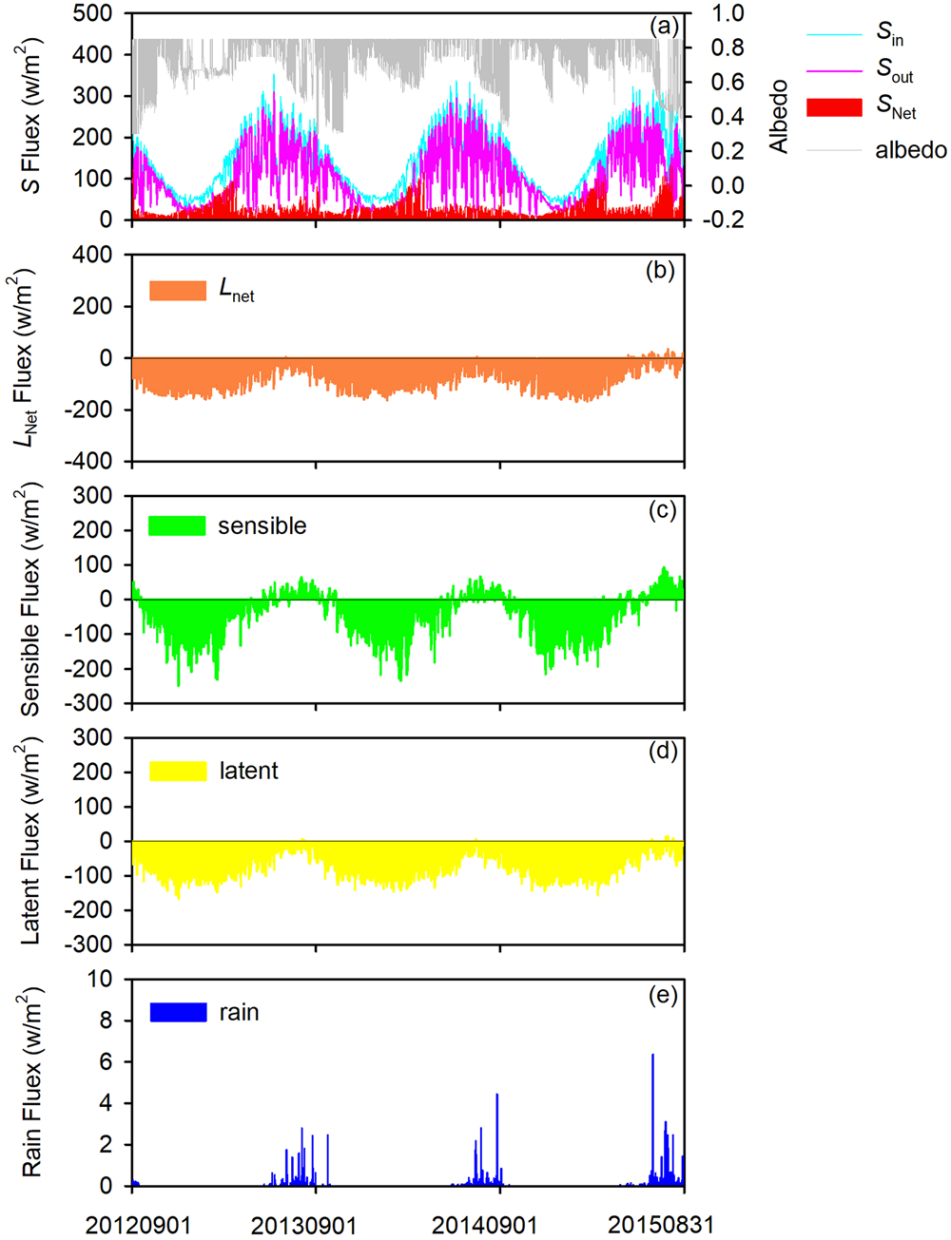
Hourly energy fluxes on the glacier surface. In plots, *S*_in_ denotes the incoming shortwave radiation, *S*_out_ denotes the outgoing shortwave radiation, *S*_net_ denotes the net shortwave radiation, and *L*_net_ denotes the net longwave radiation.

Based on outputs from the model simulating, energy balance characteristics of the URGN1 during the period of 2012–2015 were analyzed comprehensively (Fig. 5). Here, we assumed that the total energy flux was equal to the sum of absolute values of net shortwave radiation (*S*_Net_), net longwave radiation (*L*_Net_), latent heat flux (*Q*_L_), sensible heat flux (*Q*_H_), and rain sensible heat (*Q*_R_), respectively, i.e., |*S*_Net_| + |*L*_Net_| + |*Q*_H_| + |*Q*_L_| + |*Q*_R_|. We found that *L*_Net_ was accounted for 33% of the overall energy fluxes during the simulated period, which was much more than the fluxes of *S*_Net_ (13%). During the study period, there was a higher albedo (*α*) of the mean value was at 0.67, which was ranged from 0.30 to 0.875. The glacier surface could obtain the more energy fluxes of *S*_in_, but they were reflected by higher *α* from snow, firn, and ice. In addition, a lot of precipitation occurred in summer, which indicated that many events of snowfall were happened in the period. It brought the fresh snow and cloudy, which were further reflected the more solar energy fluxes due to high albedo and prevented the shortwave radiation from glacier surface, respectively (Fig. 5a,b). The energy fluxes *L*_Net_ was higher than others, and note that it was main outgoing longwave radiation. The results also pointed out that the rain fluxes was less than 1% (0.04%), which can be ignored in the simulation of glacier melting. In addition, the latent and sensible heat fluxes were accounted for 32% and 22%, respectively.

### Performance of energy-mass balance model

**Figure 6:**
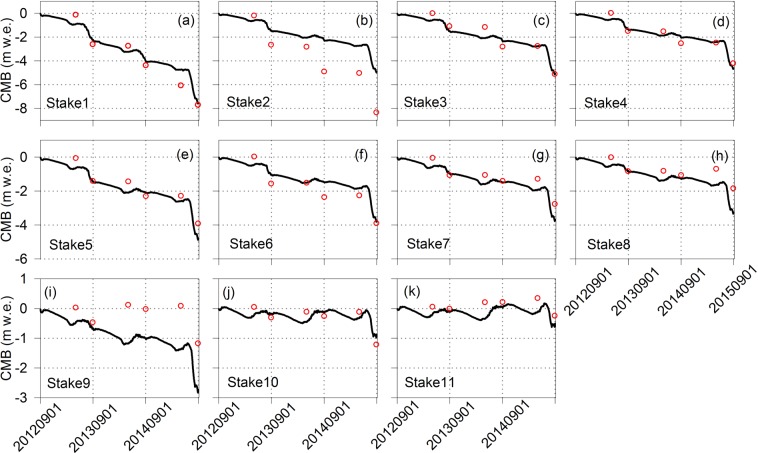
Comparison the ablation and accumulation of observation site between simulation and measurement. In plots, “CMB” denotes the cumulative mass balance, red circle denotes the measured cumulative mass balance, and black line denotes the simulated cumulative mass balance.

**Figure 7:**
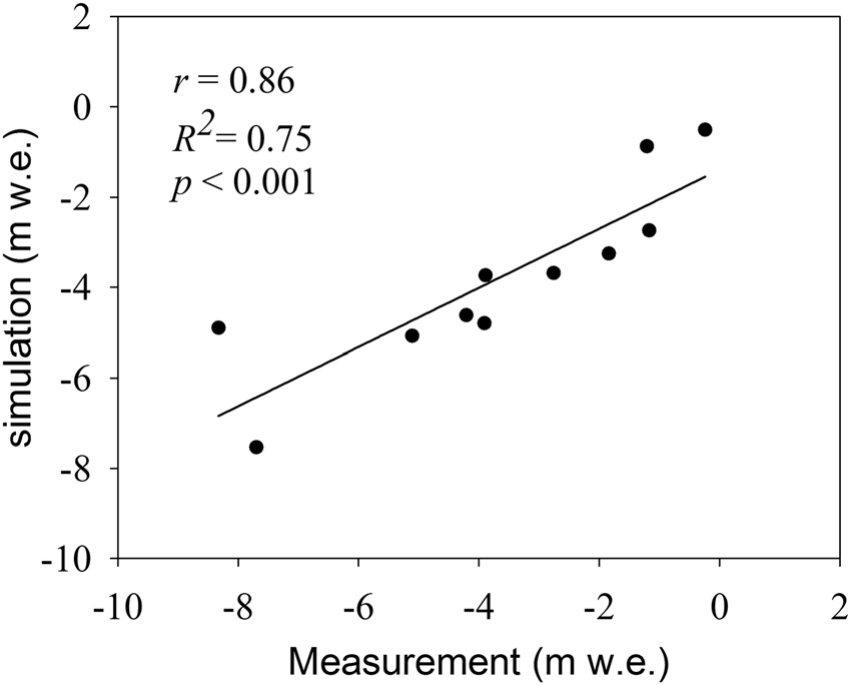
Comparison of the cumulative mass balances between simulation and measurement. In plot, *r* denotes the correlative coefficient, *R*^2^ denotes the coefficient of determination, and *p* denotes the significant level of the statistic test.

The energy mass balance model was forced from September 1st in 2012 to August 31th in 2015 by one hour step. To make a stable of the model, it was run again and again after each calibrating until the output very closest to the measurements. The simulations of 11 gridcells, corresponding to 11 validation stakes of East URGN1, were output to validate the performance of the model in different position. During the study period, the stakes and snow pits in URGN1 were measured twice per year, including early May and early September corresponding at the end of accumulation period and ablation period, respectively. The results showed that simulations of these grids were performed well except for the stakes 2 and 9 from Sep 1st 2012 to Aug 31st 2015 (Fig. 6). The simulations in accumulation period (i.e. winter) were weaker than that in ablation period (i.e. summer), which mainly resulted from the redistribution of snowpacks by wind-blowing. In addition, to further assess the final simulation of whole glacier, we also compared cumulative mass balance of the whole glacier between the simulation and measurement. The relative coefficient (*r*) between simulation and measurement in terms of cumulative mass balance was 0.86, and the coefficient of determination (*R*^2^) of 0.75 (Fig. 7). Overall, the model had good skill at simulating the processes of mass balance of URGN1 during the study period.

**Figure 8:**
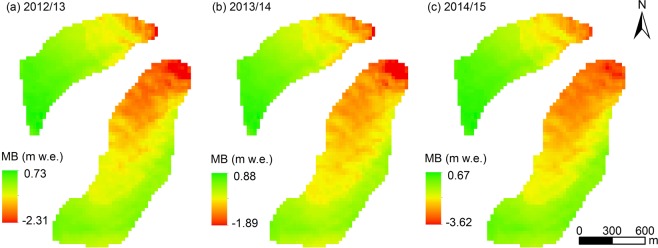
Spatial distribution of simulated mass balance of URGN1 during the periods of 2012-2013, 2013-2014, 2014-2014, respectively. In plots, the MB denotes the annual mass balance.

As shown in Fig. 8, simulated spatial distribution of mass balance was perfected and reasonable, which was in line with actual observation^35^. The maximum mass loss of URGN1 occurred in glacier front, which was decreased with increasing in altitude. The glacier ablation and accumulation were also affected by the glacial topography, significantly. For eastern branch glacier, the melting in western regions was stronger than that in eastern regions with the higher elevation and steeper (Fig. 8), because there were covered by mountain shadow (Fig. 1). In space, annual mass balance ranged from 0.73 m w.e. to −2.31 m w.e. for 2012/13, from 0.88 m w.e. to −1.89 m w.e. for 2013/14, and from 0.67 m w.e. to −3.62 m w.e. for 2014/15, respectively. Spatial distributions of simulated mass balance were in line with the observation in URGN1, whereas, the annual total error of simulation was at 3.35% in terms of annual net mass balance. The energy-mass balance performed very well in terms of simulating glacier-melt process of URGN1. In a sum, this model can be used to explain the respond of glacier mass balance to climate change.

### Sensitivity of glacier mass balance to meteorological condition

Sensitivity of glacier mass balance mainly depends on input climate data, especially the specific climate mechanism of the reference run. To assess the climatic sensitivity of glacier mass balance, perturbation experiments were conducted in this paper. The model was forced by meteorological datasets from AWS1 during the period of 2012–2015 and regarded as a reference run. Air temperature and precipitation were essential for almost glacier models, which were ofthen used to understand the climatic sensitivity of glacier mass balance. In this paper, therefore, we simulated the mass balance sensitivity by changing the air temperature of using fixed steps for the model entry, i.e., changes of ±0.5 K in air temperature, while the precipitation input changed by ±10% in total precipitation. Meanwhile, other variables unchanged. In addition, air temperature and precipitation were both changed at the same time to force the model (i.e., a coupled parameter perturbation), which mainly answered to how much precipitation can be compensated to the additional glacier mass loss due to increasing by 1 K in air temperature.

**Figure 9:**
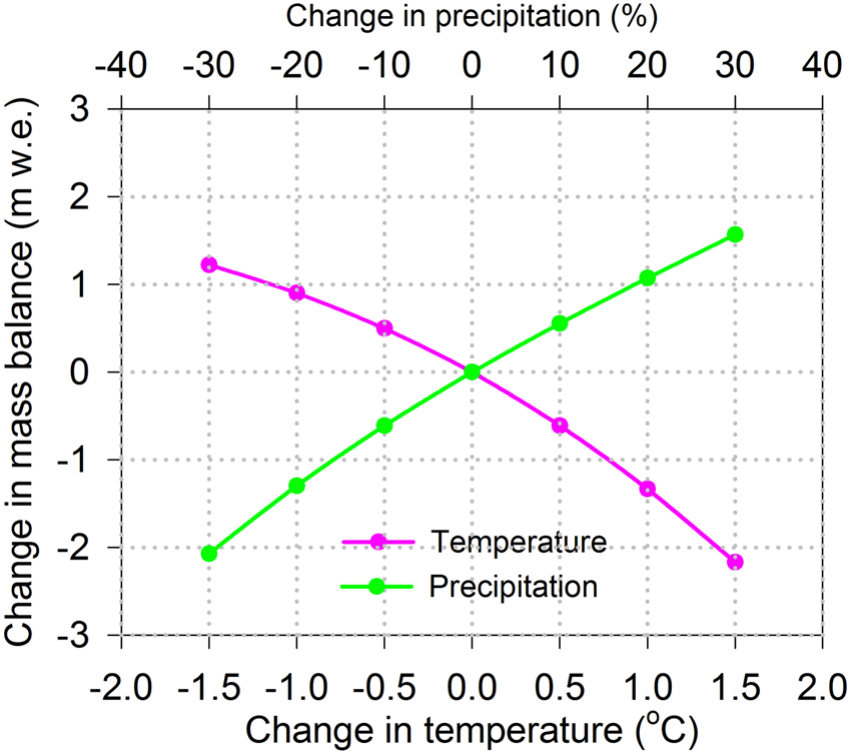
Simulating response of mass balance in the URGN1 to the air temperature and precipitation.

The simulations of six temperature-change scenarios were designed by perturbing the air temperature by a step of 0.5 K, with increasing by 0.5 K from −1.5 K to +1.5 K, and other input parameters unchanged. The results showed that total of mass balance of URGN1 was decreased by 39.95% (i.e., mass loss increased) with increasing temperature of 0.5 K, while it was increased by 32.74% with decreasing temperature of 0.5 K (Fig. 9). Total mass balance was decreased by 142.44% when the air temperature increased to 1.5 K, while it was increased by 80.47% due to decrease of 1.5 K. We also conducted the simulations of six scenarios about precipitation-change, i.e., the precipitation was changed by a step of 10%, ranging from −30% to +30%. The total of mass balance was increased by 0.55 m w.e. (36.47%) with an increase of 10% in precipitation (i.e., glacier mass input increased), while it was decreased by 0.61 m w.e. (40.16%) with a decrease of 10% in precipitation. As increasing in precipitation to 30%, the total mass balance was positive of 0.05 m w.e. (i.e., glacier mass input increased by 103.13%). We also found that the rate of glacier mass loss was accelerated as increasing in air temperature, while the compensated effect of glacier mass from the increasing precipitation was gradually limited (Fig. 9). Besides, the experiment of changes in both of air temperature and precipitation was also done. The simulations presented that the precipitation needed to be increased by 23% in order to compensate to additional mass loss resulted from increasing by 1 K in air temperature for URGN1.

The sensitivities of glacier mass balance to meteorological condition, e.g., air temperature and precipitation, were widely conducted using glacial models^34,35,46–48^. The sensitivity of mass balance to other meteorological component was also crucial to explain the relationship of glacier melting and climate change, such as, shortwave radiations (*S*), wind speed (*u*), and relative humidity (*RH*). In the paper, the perturbed value were set to the ±50 W/m^2^ for *S*_in_, ±1.1 m/s for *u*, and ±11% for *RH* according to the characteristics of meteorological factors and other implemented simulations^46,49–52^. Based on running of the glacier model, the results showed that the annual mass balance was decreased by 0.147 (−9.66%) m w.e. when increased by 50 W/m^2^ in *S*_in_ (+28.81%), while it was increased by 0.114 (7.49%) m w.e. when decreased by 50 W/m^2^ in *S*_in_. For the *u*, the annual mass balance was decreased by 0.367 (−24.11%) m w.e. (increased by 0.487 m w.e., according for 31.99%) when increased by 1.1 (+54.04%) m/s (decreased by 1.1 m/s). For the *RH*, the annual mass balance decreased by 0.131 (−8.61%) m w.e. (increased by 0.119 m w.e., according for 7.82%) when increased by 11% (+21.41%) (decreased by 11%).

## Discussion

There were significantly increased trends in annual air temperature and annual precipitation in the study region during the period of 1958–2015, with increasing rates of 0.25 °C/decade and 18.0 mm/decade (Fig. 2), respectively. The standard deviations for annual air temperature and annual precipitation were 0.63 °C and 71.50 mm (15.49%), respectively. According to the sensitivity analysis (Fig. 9), the corresponding biases for mass balance resulted from the air temperature and precipitation variability were ~0.10 m w.e and ~0.87 m w.e., respectively. In addition, the air temperature increased by 1.45 °C from 1958 to 2015, while the precipitation increased by 104.40 mm (i.e. increased by 22.08% based on the annual precipitation in 1958). If simply calculated, we found that the mass loss from increasing in air temperature was significantly higher than that from compensating in precipitation. Therefore, we deduced that the glacier mass loss in East Tien Shan was mostly resulted from increasing in air temperature, and this trend will be lasted under this climate pattern.

One of the main finding of our work was that the model was most sensitive to parameters controlling the *α*_snow_ and t^*^, which directly decided how much incoming shortwave radiation was absorbed by the glacier surface. Net shortwave radiation on the glacier surface, which was mainly energy fluxes in terms of glacier directly melting. For the Guliya Ice Cap in the West Kunlun Mountains, however, the most energy available for surface ablation was latent heat flux, while the incoming energy supplied by net shortwave radiation was almost balanced by the energy loss through net longwave radiation. For the meteorological conditions, they were different that the changes of mass balance resulted from increasing or decreasing in input element (Table 1), which indicated that the respond of glacier mass balance to climate change was non-linear. The theoretical calculation presented that glacier melt increased by 0.236 m w.e. when the *T*_a_ increased by 1 K at the observed site of meteorological station on Haig Glacier of Canadian Rocky Mountains^24^. The sensitivity of annual mass balance for URGN1 was at −0.44 m w.e. and 0.30 m w.e. when increasing and decreasing air temperature of 1 K, respectively. Note that the variation in mass balance with changing in air temperature showed the non-linear pattern in the study (Fig. 9). The sensitivity of Laohugou Glacier No.12 in Qilian Mountains of northeastern Tibetan Plateau was higher, i.e., the total mass balance of decreased by 0.40 m w.e. and increased by 0.47 m w.e. when *T* increased and decreased by 0.5 K, respectively^52^. In addition, the mass balance of Guliya Ice Cap was decreased by 0.50 m w.e. when increasing by 1 K in air temperature^46^, while the sensitivity was 1.28 m w.e.K^−1^ to Parlung Glacier No.4 in southeast Tibetan Plateau, 1.30 m w.e.K^−1^ to Zhadang Glacier in southeast Tibetan Plateau, and 0.18 m w.e.K^−1^ to Muztag Ata Glacier No.15 in Muztag Ata region^51^. Besides, the sensitivity of glacier annual mass balance to 1 K in air temperature was ranged from 0.2 m w.e. to 0.5 m w.e. in Tien Shan Mountains^34,53^, while one study^54^ found it was ranged from −0.63 to −0.72 m w.e. when increasing 1 K in air temperature for the individual drainage basins in Switzerland. The simulated work^34^, using the simple Degree-Day Model, which presented that the mass balance sensitivity of URGN1 was 0.48 m w.e.K^−1^. Thus, the assessment of mass balance sensitivity was in a reasonable range from 0.38 m w.e. K^−1^ to 0.56 m w.e. K^−1^ ^55,56^. Sensitivity of the glacier mass balance to air temperature was lower than that of Laohugou Glacier No.12, Guliya Ice Cap glacier, Parlung Glacier No.4, and Zhadang Glacier, while it was higher than that of Muztag Ata Glacier No.15 and the theoretical calculation for Haig Glacier of Canadian Rocky Mountains.

**Table 1:** Sensitivities of glacier mass balance to the shortwave radiations (*S*), wind speed (*u*), and relative humidity (*RH*).

Perturbation	Changes in terms of the mean hour value	mass balance change (m w.e./a)		Sensitivity
		Total change	Mean annual change	
*T*	+1 K	−1.33 (−87.58%)	−0.44 (−29.19%)	−0.44 m w.e.K^−1^
*T*	−1 K	0.90 (59.28%)	0.30 (19.76%)	0.30 m w.e. K^−1^
*P*	+10%	0.55 (36.47%)	0.18 (12.16%)	0.055 m w.e.%^−1^
*P*	−10%	−0.61 (−40.16%)	−0.20 (13.39%)	−0.061 m w.e.%^−1^
*S*_in_ + 50 W/m^2^	+28.81%	−0.443 (−28.99%)	−0.147 (−9.66%)	−0.015 m w.e.%^−1^
*S*_in_ − 50 W/m^2^	−28.81%	0.343 (22.48%)	0.114 (7.49%)	0.012 m w.e.%^−1^
*u* + 1.1 m/s	+54.04%	−1.101 (−72.34%)	−0.367 (−24.11%)	−0.020 m w.e.%^−1^
*u* − 1.1 m/s	−54.04%	1.461 (95.99%)	0.487 (31.99%)	0.027 m w.e.%^−1^
*RH* + 11%	+21.41%	−0.393 (−25.83%)	−0.131 (−8.61%)	−0.018 m w.e.%^−1^
*RH* − 11%	−21.41%	0.358 (23.47%)	0.119 (7.82%)	0.017 m w.e.%^−1^

The sensitivity of mass balance to precipitation were 0.055 m w.e.%^−1^ for increasing precipitation and −0.061 m w.e.%^−1^ for decreasing precipitation in URGN1, respectively, i.e., the mass balance was increased by 0.18 (12.16%) when precipitation increased by 10%. The mass balance sensitivity to precipitation of +10% were increase of 0.29 m w.e. for Parlung Glacier No.4, 0.52 m w.e. for Zhadang Glacier, and 0.21 m w.e. for Muztag Ata Glacier No.15, respectively^51^. For the Guliya Ice Cap glacier, mass balance was decreased by 0.38 m w.e. when precipitation decreased by 20%. The precipitation sensitivity was ranged from +0.03 m w.e. and +0.44 m w.e. when increased precipitation by 10%, whereas the humidity was also an important factor to affect mass balance sensitivity^54^. The precipitation, including the liquid and solid precipitation, was difficult to accurately capture and separate in mountain regions. The solid precipitation/snowfall directly input to glacier mass, which usually dealt with some different ways. For instance, in simulating of Laohugou Glacier No.12, the solid precipitation was restricted within 10% of the annual mass balance^52^, while other work used the threshold temperature or a ranged temperature, for example, 1 °C was used to simulate of Rhonegletscher glacier in the central Swiss Alps^32^, 2 °C was widely used in the Chinese Tien Shan^57^, and the air temperature thresholds for rain and snow were 2.3 °C and −0.5 °C on the Parlung Glacier No.94 in southeast Tibetan Plateau, respecitivitly^50^. One study relative to separate methods of precipitation types in China’s region^58^, which showed that precipitation types were highly dependent on the surface elevation and humidity, whereas the separated air temperature of rain and snow ranged in the different regions.

Similarly, there were different sensitivities of mass balance to the *S*_in_, *u*, and *RH*, respectively. Based on the theoretical calculation of glacier mass balance model^24^, a 10 W/m^2^ increased in *S*_in_, a 1 m/s increased in *u*, and 12–79% (i.e., increased by 1 g/kg in *RH* and fixed temperature) increased in *RH* resulted in an increase of 0.15 m w.e, 0.05 m w.e., and 0.08 m w.e. (3%) (i.e., summer melt) in mass balance, respectively. In this paper, our simulation showed that the mass balance sensitivity was at −0.020 m w.e.%^−1^ and 0.027 m w.e.%^−1^ to increase and decrease of 1.1 m/s in *u*, respectively. The sensitivity of mass balance to *u* change (±1.1 m/s; according for ±54.04%) was higher than that to both *S*_in_ (±50 W/m^2^; according for ±28.81%) and *RH* (±11%; according for 21.41%) change in URGN1. In the simulation of mass balance for Parlung Glacier No.94, which also found that the sensitivity of mass balance to *u* change (±1.1 m/s) was higher than that of changes in *S*_in_ of ±46.4 W/m^2^ and *RH* of ±11.3%^45^. The sensitivity of mass balance to *S*_in_ was lower than that to *u* and *RH*, which mainly resulted from the net shortwave radiation fluxes according for relative small part in total of energy fluxes on the glacier surface. For Laohugou Glacier No.12 in Qilian Mountains, the mass balance decreased to 8.27 m w.e. when mean *S*_in_ increased by 20% (about 24.8 W m^2^), i.e., approximately twice larger than that caused by 10%^52^. In other word, the mass balance of Laohugou Glacier No.12 was very sensitivity to *S*_in_. The mass balance of Guliya Ice Cap, however, would only be positive when *S*_in_ reduced by at least 19.5% (12 W m^2^) under the scenario of decreasing by 1 °C in air temperature and increasing by 69% in precipitation at the same time^46^.

In summary, the glacier mass balance in different mountain showed the differing sensitivity due to the local climatic conditions. The different sensitivity was mainly resulted from the discrepancies in the ratio of snowfall to precipitation during the ablation season^51^, the amount of melt energy during the ablation season, precipitation seasonality, and spatial difference of topography among the different local regions. The interactions reflected that the sensitivity of glacier mass balance in response to climate change mainly resulted from the different local condition of climate change and physical characteristics of local glacier^34,50,53,54^.

## Methods

### Meteorological data

Since 1958, the changes in meteorological conditions at the headwater of Urumqi River recorded by Daxigou meteorological station (i.e., AWS2 in Fig. 1). AWS2 located in 3 km southeast of Urumqi River Glacier No.1 and was installed by China Meteorological Administration in 1958. In the paper, the meteorological dataset of AWS2 were used to understand the climate change in the region during the period 1958–2015 (Table 2), which derived from the online http://data.cma.cn/. The accumulation and ablation from September to next August are used to describe the changes of one hydrological year, therefore, the period of September to next August also regard as one year of mass balance and climate change in this paper.

**Table 2:** Information of meteorological stations in the paper.

Sits	Name	Longitude (°)	Latitude (°)	Elevation (m a.s.l.)	Period (year)	Data source
AWS1	Observation No.1	86.81	43.12	3840	2012–2015	In this study
AWS2	Daxigou	86.84	43.11	3539	1958–2015	http://data.cma.cn/

Note: *T* denotes the air temperature, *P* denotes the precipitation.

### Energy balance model

The energy balance model all components of the radiation balance, the sensible heat flux and the latent heat flux are estimated at each time step of each gridpoint using climate and glacier records. The energy available for melt is calculated as the residual term in the energy balance equation. Energy fluxes directed towards the glacier/snow surface are defined as positive. The mass balance model is described as the following equation^59^:

1 $${Q}_{M}=G(1-\alpha )+{L}_{Net}+{Q}_{H}+{Q}_{L}+{Q}_{G}+{Q}_{R}$$

where *G* denotes the global radiation, which expressed as the incoming shortwave radiation in this paper (measured by AWS1). *α* is the surface albedo. *L*_*Net*_ is the longwave radiation balance, which is calculated by incoming minus outgoing longwave radiation. *Q*_*H*_ is the sensible heat flux. *Q*_*L*_ is the latent heat flux. *Q*_*G*_ is the ground heat flux which will be ignored in this paper, and *Q*_*R*_ is the sensible heat supplied by rain. *Q*_*M*_ is the melt energy at the each gridcell.

(1) Surface albedo (*α*)

Surface albedo is critical to the glacier surface energy balance, especially the albedo of snow. Snow albedo ranged from snow to firn, and to ice. Here, the snow albedo is computed according to the parameterization of Oerlemans and Knap^60^ for each time step by

2 $${\alpha }_{snow}={\alpha }_{firn}+({\alpha }_{frsnow}-{\alpha }_{firn})\,\exp \,(\frac{s-i}{{t}^{\ast }})$$

where *α*_*firn*_ is the albedo of snow below fresh snow (0.6), *α*_*frsnow*_ is the albedo of fresh snow (0.875), t^*^ is the time scale determining how fast the snow albedo approaches the albedo of firn after a snowfall (21.9 days), *s* is the number of the day on which the last snowfall occurred.

Albedo is adjusted to allow for smooth transition to ice albedo if snow depth (d) is small:

3 $${\alpha }_{snow}={\alpha }_{snow}+{\alpha }_{ice}-{\alpha }_{snow}\,\exp \,(\frac{-d}{{d}^{\ast }})$$

where d^*^ is a characteristic scale for snow depth (3.2 cm), *α*_*ice*_ is the albedo of ice surface (0.3). When snow depth equals to the d^*^, the snow cover contributed 1/e to the albedo and the underlying surface (1–1/e).

(2) Longwave radiation

The longwave incoming radiation (*L*↓) and longwave outgoing radiation (*L*↑) can be obtained from direct measurement at the climate station on the glacier surface. However, the meteorological station is generally installed on the rock or in the non-glacier region, and keeping special distance from station to glacier surface. Then, the *L*↑ from glacier surface can be calculated by the Stefan-Boltzmann law from surface temperature (*T*_0_) and surface emissivity (*ε* equals to 1):

4 $$L\uparrow =\sigma \varepsilon {({T}_{0}+273.15)}^{4}$$

where *σ* is the Stefan-Boltzmann constant (5.67 × 10^−8^ W m^−2^K^−4^). In this paper, the longwave radiation was measured by AWS1, and the temperature of glacier surface was assumed to 0 °C (−315.6 W/m^2^).

(3) Turbulent heat fluxes

The sensible heat flux (*Q*_*H*_) is calculated as a function of air temperature (*T*) and wind speed (*u*) at 2 m^61^ by

5 $${Q}_{H}=A\ast T$$

A is the transfer coefficient as defined by:

6 $$A=5.7\sqrt{u}$$

The latent heat flux (*Q*_*L*_) is calculated as a function of humidity and wind speed by

7 $${Q}_{L}=A\ast 0.623\ast \frac{{L}_{v}}{p\ast {c}_{p}}(e-{e}_{0})$$

where the *L*_*v*_ is the latent heat of evaporation (2514000 J/Kg). *c*_*p*_ is the specific heat capacity (1005 J/(kg·K), *p* is the atmospheric pressure, *e*_0_ is the saturation vapour pressure of melting ice (611 Pa), and *e* is the vapour pressure.

(4) Other heat fluxes

The energy supplied by the sensible heat of rain is approximated by

8 $${Q}_{{\rm{R}}}={c}_{\omega }R\ast ({T}_{{\rm{r}}}-{T}_{{\rm{s}}})$$

where the *c*_w_ is the specific heat of water [J/kg/K], *R* is rainfall rate, *T*_r_ is the temperature of rain, and *T*_s_ surface temperature. The rain temperature is assumed to be identical to screen-level temperature.

### Snow accumulation

The value of snow accumulation (*P*_snow_) is widely known to have important implications for the glacier mass balance modeling by means of snowfall-albedo association. Snowfall is also the main input to the accumulation of Urumqi Glacier No.1. Generally, *P*_snow_ is modeled by the total daily precipitation (*P*) and two critical air-temperature thresholds for rain (*T*_L_) and snow (*T*_s_). In the Chinese Tien Shan, however, a study^56^ found that the air temperature threshold of T_0_ = 2 °C was critical, which showed well skill to distinguish between solid precipitation and liquid precipitation. We herein have adopted the air temperature threshold to distinguish the liquid precipitation (i.e. snowfall). When *T* is above *T*_0_, *P*_snow_ is equal to zero or *P*. Within the threshold value, the precipitation is solid or snow, *P*_snow_ was calculated from the model as follow:

9 $${P}_{snow}=\{\begin{array}{c}P,{T}_{a} < {T}_{0}\\ 0,{T}_{a}\ge {T}_{0}\end{array},{T}_{0}=2^\circ {\rm{C}}$$

### Water equivalent

Finally, the energy available for melt as derived from the residual term in the energy balance equation is converted to water equivalent melt (WE [mm/timestep]).

Total ablation is obtained considering melt and (re-)sublimation

10 $$W{E}_{abla}=\{\begin{array}{c}\frac{{Q}_{m}}{{L}_{f}}\ast 3600\ast timestep,\,{Q}_{L} > 0\,and\,{T}_{s}=0\,\mbox{--}\, > condensation,\,no(re)\,sublination\\ (\frac{{Q}_{m}}{{L}_{f}}-\frac{{Q}_{subl}}{{L}_{s}})\ast 3600\ast timestep,\,{Q}_{L} < 0\,or\,({Q}_{L} > 0\,and\,{T}_{s} < 0)\end{array}$$

where L_*f*_ is the latent heat of fusion (3.34 × 10^5^ J/kg) and L_*s*_ is the latent heat of sublimation (2.849 × 10^6^ J/kg).

The mass balance model is described as the following equation:

11 $$B=\int (\frac{{Q}_{m}}{{L}_{m}}+\frac{{Q}_{L}}{{L}_{v}}+{P}_{snow})dt$$

where B is the point mass balance (of gridcell in galcier) in the unit of meters water equivalent (mw.e.). *Q*_m_ is the melt energy, *Q*_L_ is the turbulent latent heat flux (associated with ice/snow sublimation or deposition), *L*_m_ and *L*_v_ are the latent heat of ice melt (3.34 × 10^5^ J/kg) and evaporation/sublimation (2.50 × 10^6^ J/kg/2.83 × 10^6^ J/kg). In the model, the refreezing of melt water in sub-glacial is not considered.
